# Common spatial pattern for classification of loving kindness meditation EEG for single and multiple sessions

**DOI:** 10.1186/s40708-023-00204-9

**Published:** 2023-09-09

**Authors:** Nalinda D. Liyanagedera, Ali Abdul Hussain, Amardeep Singh, Sunil Lal, Heather Kempton, Hans W. Guesgen

**Affiliations:** 1 School of Mathematical and Computational Sciences, Massey University, Palmerston North, 4410 New Zealand; 2 Department of Computing & Information Systems, Faculty of Applied Sciences, Wayamba University of Sri Lanka, Kuliyapitiya, 60200 Sri Lanka; 3 Universal College of Learning (UCOL), Palmerston North, 4410 New Zealand; 4 School of Psychology, Massey University, Auckland, 0632 New Zealand

**Keywords:** BCI (brain computer interface), EEG (electroencephalography), Meditation, Classification, CSP (common spatial patterns), LDA (linear discriminant analysis)

## Abstract

While a very few studies have been conducted on classifying loving kindness meditation (LKM) and non-meditation electroencephalography (EEG) data for a single session, there are no such studies conducted for multiple session EEG data. Thus, this study aims at classifying existing raw EEG meditation data on single and multiple sessions to come up with meaningful inferences which will be highly beneficial when developing algorithms that can support meditation practices. In this analysis, data have been collected on Pre-Resting (before-meditation), Post-Resting (after-meditation), LKM-Self and LKM-Others for 32 participants and hence allowing us to conduct six pairwise comparisons for the four mind tasks. Common Spatial Patterns (CSP) is a feature extraction method widely used in motor imaginary brain computer interface (BCI), but not in meditation EEG data. Therefore, using CSP in extracting features from meditation EEG data and classifying meditation/non-meditation instances, particularly for multiple sessions will create a new path in future meditation EEG research. The classification was done using Linear Discriminant Analysis (LDA) where both meditation techniques (LKM-Self and LKM-Others) were compared with Pre-Resting and Post-Resting instances. The results show that for a single session of 32 participants, around 99.5% accuracy was obtained for classifying meditation/Pre-Resting instances. For the 15 participants when using five sessions of EEG data, around 83.6% accuracy was obtained for classifying meditation/Pre-Resting instances. The results demonstrate the ability to classify meditation/Pre-Resting data. Most importantly, this classification is possible for multiple session data as well. In addition to this, when comparing the classification accuracies of the six mind task pairs; LKM-Self, LKM-Others and Post-Resting produced relatively lower accuracies among them than the accuracies obtained for classifying Pre-Resting with the other three. This indicates that Pre-Resting has some features giving a better classification indicating that it is different from the other three mind tasks.

## Introduction

As defined by Thomas et al. [1], meditation simply means targeting mental and emotional control by focusing the mind on a thought, activity or an object resulting in a clear and calm mind. Meditation is known to have multiple benefits such as depression reduction, stress reduction, anxiety reduction and as an attentional training to bring improved insight into one’s own mental activity [2]. To understand the hidden characteristics of meditation, scientific methods such as heart rate, brain waves, brain images, questioners [3–7] have been used and in this study, brain wave data collected on multiple meditation sessions using electroencephalography (EEG) were used. EEG is a test used to monitor the electrical activity of the brain and has been successfully used in studying various types of meditation [8–10]. The present work is aimed at achieving a classification for loving kindness meditation (LKM) EEG data for a variety of instances. The selected EEG dataset consists of four types of mind tasks, two meditation and two resting (one before meditation and one after meditation). The study was conducted using EEG data of 32 participants for a single session and 15 participants for 5 sessions. The analysis was done for both single session and multi-session instances. A machine learning approach was used to get the results in which Common spatial patterns (CSP) was used for feature extraction and Linear discriminant analysis (LDA) was used for classification. The classification was done for all six pairs of the four mind tasks that gave a better understanding of the LKM EEG dataset.

### EEG BCI background

In recent years, research has been conducted to develop various sensor devices that can read information from the human body and can be used for operations in the outside world. This involves a broad area where sensors may try to detect features such as heartbeat, breath, eye movement, brain waves, etc. The field in which brain waves are used for communicating with a computational device is known as brain computer interface (BCI) [11]. The brain functions by transferring information between brain cells known as neurons in the form of electrical signals and chemicals. These electrical signals at a given time indicate how a brain functions and both researchers and doctors have been using the behavior of these signals as a tool to understand the functionality of the brain. Electroencephalography (EEG) which is one such method used to measure this electrical activity in millivolts, measures the voltage changes produced from the flow of ionic current among neurons of a brain. The patterns of electrical activity occurring in the brain are known as brain waves. These brain waves are basically classified into five categories which are defined based on the frequency strength and are assigned to different activities occurring in the brain such as deep sleeping, meditating, creativity, alertness, problem solving, etc. Although there are small variations in the boundaries of these bands, the mostly used set of frequencies among researchers are Delta (0.5–4 Hz), Theta (4–8 Hz), Alpha (8–13 Hz), Beta (13–30 Hz) and Gamma (> 30 Hz) [12–14].

EEG data are collected by placing electrodes along the scalp and collecting data for a certain period of time. The international 10–20 system [15] of electrode placement is an internationally recognized numbering system used to identify locations on the scalp when placing electrodes of an EEG device on the scalp. The electrodes placement is designated by numbers and letters to represent the area of the brain underneath the electrode, Fp: pre-frontal region, F: frontal region, P: parietal region, T: temporal region, O: occipital region and C: central region [16–18]. To represent electrodes placed on the boarder of any of the two regions, the letters of both regions are used [19, 20] (FC/frontal-central region, FT/frontal-temporal region, CP/central-parietal region, TP/temporal-parietal region and PO/parietal-occipital region) and AF is used between Fp and F [21]. The numbers represent the position on the scalp with odd numbers for the left hemisphere and even numbers for the right hemisphere. When collecting EEG data any number of electrodes can be used, placed in any pattern in international 10–20 system and in most of the studies an electrode count of 4, 8, 16, 32, 64, 128 or 256 has been used.

### EEG signal processing with machine learning techniques

EEG data contain a variety of information about the brain activity for a given instance and pattern extraction and deriving information from them are complex processes. This is basically due to the fact that the raw EEG data can contain high levels of noise (low signal-to-noise ratio) and thus it needs to follow multiple steps to process the signals to extract the valuable information hidden in the raw EEG data. The steps involve data acquisition, signal preprocessing and artifact removal, feature extraction, classification, and control interface [22]. When considering signal preprocessing and artifact removal, there are two types of noise in EEG data, and these are the noise added from the external environment such as the electromagnetic effects from surrounding electrical equipment and the noise comes from various bodily activities such as eye movements, eye blinks, jaw movements, etc. Here, usually a suitable filtering technique can be used to remove noise coming from internal body functions and a high pass filter [9] can be used to remove unwanted lower frequencies such as frequencies less than 0.5 Hz. A low pass filter [9] can be used to remove unwanted higher frequencies such as frequencies greater than 60 Hz. To remove the external electromagnetic noises, 50 Hz band pass filter [23] can be used.

For signal processing and feature extraction from EEG data, sometimes advanced algorithms such as common average reference (CAR), independent component analysis (ICA), principal component analysis (PCA), Fourier transformation (FT), linear discriminant analysis (LDA), common spatial pattern (CSP) can be used depending on the problem type [24–26]. In CAR, to reduce the noise, the average value of all the electrodes is removed from all the electrodes. On the other hand, ICA is a special case of blind source separation, an example is “the cocktail party problem” which deals with the problem of distinguishing a person’s voice in a noisy room. When removing artifacts, ICA [27, 28] will try to separate the artifacts into independent components from the EEG signals using the data characteristics. Similarly in feature extraction, ICA will try to detect the influence creating on each other among the channel data and tries to reduce it so that the channel data will be independent from one another. This is done by thinking that each channel data is a non-Gaussian signal, and they are statistically independent from one another.

Principal component analysis (PCA) [28, 29] tries to reduce the number of variables in a dataset. When doing this task, some information may get lost in the dataset. So, PCA tries to reduce the data size while losing minimum information as possible. This size reduction allows various machine learning algorithms to work efficiently. In EEG data processing, PCA can be used not only for cleaning the data, but also for extracting features.

Fourier transformation (FT) [30, 31] can be seen as a feature extraction method used in many EEG-related experiments. FT is a mathematical transformation of data that will convert a vibration signal into its frequency domain. So, the result will show how each frequency has contributed to the original vibration signal.

Common spatial pattern (CSP) [32–35] is a multivariate signal processing method used in EEG feature extraction methods that tries to maximize the variance of one mind task while trying to minimize the variance of the other mind task. The idea is to identify a set of spatial filters that will maximize the variance between two mind tasks in EEG. The algorithm starts by dividing the dataset based on the two mind tasks and then calculate a covariance matrix for each mind task. After that a pooled covariance matrix is calculated using all the covariance matrices. Then, the spatial filters that give a maximum covariance difference between the two mind tasks are calculated using generalized eigen vectors obtained from the pooled matrix. These spatial filters are sorted in the descending order of eigen values obtained from the pooled covariance matrix. After that, each mind task EEG dataset is passed through the spatial filters to get the spatially filtered features for each mind task. These features get ranked according to their strength of separating the two mind tasks and the strongest features will be used in classifying a new EEG dataset.

With CSP as a feature extraction algorithm, linear discriminant analysis (LDA) [36, 37] as a classification algorithm has been used, especially in the area of motor imagery BCI. LDA is a machine learning algorithm that will either calculate or use an already built pooled covariance matrix to calculate the feature set, which in other words, when using the algorithm alone, LDA itself will calculate a feature set for the classification model. However, when the feature set calculated by CSP is available, LDA can take it as an input and directly use that for the classification model. Lotte et al. in his review paper conducted a study regarding classification algorithms for EEG-based brain–computer interfaces [24] and gives a summary of all the methods and algorithms used under BCI for feature extraction and classification. Among the above-mentioned algorithms, CSP is one of the best performing feature extraction algorithms for EEG in motor imagery. As shown in another review paper based on motor imagery EEG [22], the classification algorithm LDA has produced high performance in motor imagery BCI. Although this combination of CSP and LDA has been used to analyze EEG data in many experiments such as motor imagery, this machine learning pair has not been used for studying meditation EEG datasets for multiple sessions. The lack of research for using CSP and LDA to study meditation EEG is well demonstrated in a review paper [38].

### Study of meditation using EEG

Effects of meditation on humans can be divided into two types, namely state and trait while EEG has been used to study both cases [20, 39]. States are temporary changes and traits are long-term/permanent changes happening in a person doing meditation. To identify state [40, 41] characteristics, a person needs to collect data for meditation and non-meditation instances either for the same group of people or for a meditation group and a control group. The brain wave characteristic difference between meditation and non-meditation instances is one example of state changes in the brain. Trait [4, 42] changes are permanent modifications which are visible in a person after practicing meditation for a long time. Trait characteristics can be identified by comparing brain functionality of long-term meditators with non-meditators/novice meditators. This can be detected when they are staying relaxed without meditating or while doing meditation. Studies have been conducted comparing different instances of the same meditation technique such as meditation vs non-meditation, novice meditators meditating vs expert meditators meditating, using EEG data [43].

Although there are many meditation techniques, past research shows that they are categorized in to either 2 or 3 different types of groups based on the characteristics they possess. Cahn et al. [20] divide meditation into two types, mindfulness and concentrative depending on how the person focuses his/her attention in meditation. Some meditation techniques fall into either one of these two techniques or somewhere in the middle where it will have a portion of characteristics from both mindfulness and concentrative methods. Mindfulness meditation simply allows any type of emotion, thought or a feeling to appear and disappear from mind without any forceful control over it. The person will be an observer looking at the mind without any attachment, dislike or analysis and some examples are Vipassana [6, 44, 45] and Zen [46, 47] meditations. On the other hand, in concentrative meditation a person will have a special focus or an attention towards a specific mental task. This can be a body sensation like breath, body movement like walk, a repeated sound (a word or a sentence know as a mantra) or imagining of a particular symbol or picture, etc. Some examples are yoga and samatha (breath, etc.) meditations [48, 49]. However, some meditation techniques such as loving kindness [50, 51] and transcendental [52, 53] will have characteristics of both mindfulness and concentrative methods. Loving kindness meditation (LKM) which is also known as Metta meditation works by generating positive thoughts such as kindness, love, compassion towards oneself or others without getting too attached to anything. This can be achieved by repeating a phrase or couple of phrases that may wish someone to be happy or wish someone to be free from suffering. Research shows that LKM has many benefits that improves physical and mental health such as reducing depression, stress and even improving the immunity of the body [50].

### Summarizing the importance of this study

Scientifically understating how a brain works when doing meditation is an ongoing research trend in the field of meditation. Studies show that various characteristics were identified in EEG signals that map with various characteristics of meditation [1, 20, 54, 55]. As an example, when considering EEG data collected for a group of people when meditating and when not meditating, analyzed EEG data show pattern difference between the two groups [56] Moreover, there are studies that discuss about multiple benefits gained from practicing meditation [57]. Some studies show how practice of meditation supports in improving mental health [51, 58]. However, it is important to have a proper guidance for the meditation. Brandmeyer et al. [59] talks about the need of some devices/programs that can indicate how well a person is performing in meditation. The author indicates, with such facilities a person can monitor his/her progress in mediation and alter the practice according to the progress. Therefore, it has been emphasized that there is a need for monitoring tools in the research community. There have been scientific studies to understand the usefulness of apps that support meditation [60–62]. These studies show that these apps offer many benefits for the people using them [63, 64]. However, the biggest disadvantage of most of the apps available for meditation support is that the apps are not capable of knowing if the person is properly progressing in the meditation [65]. Therefore, the support and guidance an already available app can offer a person is highly limited. Taken together, it emphasizes the importance of classifying meditation/non-meditation EEG data with high accuracy and more importantly, to study if the classification of meditation/non-meditation is possible for multiple sessions using EEG data. This can result in developing algorithms that can understand various stages in meditation, hence can guide a person in meditation. Recent studies have shown the results of classifying EEG meditation/non-meditation data. Ahani et al. [9] has classified mindfulness meditation EEG data using Stockwell transform and support vector machine with an accuracy of 85.0%. At the same time, Tee et al. [66] achieved a classification accuracy of 96.9% for theta healing meditation using discrete wavelet transform and logistic regression. However, for LKM such results have not been reported so far. In both cases, the studies were conducted only for a single session EEG data. It is well understood that a study of multiple session classification is needed. Therefore, this study was done for loving kindness meditation for multiple sessions and CSP was used in feature extraction, and this is a significantly new approach.

## Methods

### Dataset description

For the current study, an online EEG dataset relevant to LKM was chosen which has been titled as “The Effect of Buddhism Derived Loving Kindness Meditation on Modulating EEG: Long-term and Short-term Effect”. The dataset has been publicly available since 2021–09-24 onwards. The dataset was collected by the team of Ven. GoonFui Wong, Junling Gao and Rui Sun with a funding of Faculty Research Fund of Faculty of Education in HKU. The project has been ethically approved by the University of Hong Kong Human Research Ethics Committee under the reference number: EA210145. The dataset DOI is "https://doi.org/10.18112/openneuro.ds003816.v1.0.1".

Brainvision—ActiCHamp (Brain Products, German) EEG device was used for the data collection. The device has 127 EEG channels placed on the international 10–20 system and one ECG channel for collecting the data [21]. To keep a good signal-to-noise ratio, all electrodes impedance was kept under 20 kOhm and the sampling rate was 1000.

EEG data have been collected using 48 participants having previous experience in meditation and were used for the two main mind tasks: meditation and non-meditation separately. Out of these 48 participants, 15 participants were used in multiple session data collection. For these 15 participants, 8–10 data collection sessions were conducted within a 2-month period. In each session, the data were collected for each person for six types of tasks with eyes closed. The meditation method used was loving kindness meditation (LKM). The six tasks were pre-resting, post-resting, radiating LKM to self, radiating LKM to others, visualize self and visualize others.

In this analysis the interest is to classify rest state EEG data with meditation EEG data. For that, EEG data of four mind tasks out of the six available tasks were selected. The selected four instances were pre-resting, post-resting, radiating LKM to self, radiating LKM to others where the first two are resting instances: one before the meditation sessions and the other after finishing all the meditation sessions. LKM to self and LKM to others are two types of meditations where a person meditate loving kindness thoughts to oneself and to others, respectively. In the current work, the labels Pre-Resting, Post-Resting, LKM Self and LKM Others are used for them.

The original dataset consists of 6467 files having a size of 53.97 GB and with 48 total number of participants from which 15 were used in multiple session data collection. With these details, two types of studies were conducted for the given dataset. One is to study the meditation/non-meditation EEG data for 48 participants for a single session while the other is to study the meditation/non-meditation EEG data for 15 participants for multiple sessions.

When considering the original dataset, each EEG data collected for a single instance (single mind task) has produced 6 files. (A single data collection session consists of six of these mind tasks (instances), which means 36 files for a single data collection session.) One out of the six files for each instance contains the raw EEG data and the other five files contain supporting data for the raw EEG data. In the original dataset a sample of those six files, for an instance called ‘aaa’ were labeled as: aaa_eeg.eeg, aaa_eeg.json, aaa_eeg.vhdr, aaa_eeg.vmrk, aaa_channels.tsv, aaa_events.tsv. To read EEG data for a single instance, the corresponding EEG file and the supporting files were used together.

### Methods of experiment

As the first step, readability of both the raw data and supporting files were checked. A very few data collection instances, in which one or more of the six needed files were corrupted, the entire session was removed from our analysis. In this study, a person/session was selected only if all four mind tasks were not corrupted. The analysis was conducted using the python language and the mne package in python was used to read the raw data using read_raw_brainvision. Out of 48 participants that can be considered for a single session, there were 32 people whose files were readable without being corrupted, for all 4 instances. Therefore, for the single session analysis raw data of these 32 participants was used.

Similarly, the mne package was used to read raw data for the 15 participants where data were collected in multiple sessions consisting about 8, 9 or 10 sessions. For these 15 participants, it was observed that 5 to 9 sessions were properly readable (for a selected good session, EEG data were properly readable for all four instances it has.). Therefore, when studying EEG data for multiple sessions, the data of five sessions (Table 1) were used for these 15 participants to allow fairness among all 15 participants. When there were more than 5 good-quality sessions available, the 5 sessions with the largest file sizes were selected and used in this study.

**Table 1:** The five sessions selected for each of the 15 participants when in multiple session analysis

Count	Participant no.	Session numbers				
1	1	1	7	8	9	10
2	3	1	2	5	6	10
3	7	1	2	3	8	10
4	8	1	4	5	6	8
5	11	2	3	4	9	10
6	12	1	2	3	4	8
7	14	1	3	5	6	8
8	37	3	4	7	9	11
9	38	1	2	3	7	10
10	39	1	5	6	7	9
11	41	3	5	7	8	9
12	42	1	3	6	7	10
13	43	1	2	3	4	5
14	44	1	3	4	6	7
15	45	1	5	7	8	9

In summary, using the loving kindness meditation EEG dataset (Pre-Resting, Post-Resting, LKM Self and LKM Others) two studies were conducted using the available readable data. In the first study, EEG data for 32 participants involved with a single session were used. For the second study, EEG data for 15 participants collected in 5 sessions were used. As the next step, cleaning of this readable raw EEG data is elaborated below.

The raw data consist of 128 channels from which one was an ECG channel. The ECG channel was removed, and the remaining 127 EEG channels were taken. Under the 10–20 montage, 4–5 channels were renamed accordingly to match the montage ‘standard_1005’. The dataset was filtered using a high pass filter of 0.5 Hz and a low pass filter of 45 Hz that gave the cleaned EEG dataset a frequency range starting from 0.5 Hz to 45 Hz. This frequency range contains 5 frequency bands that are used in studying meditation EEG; Delta (0.5–4 Hz), Theta (4–8 Hz), Alpha (8–13 Hz), Beta (13–30 Hz), Lower Gamma (30–45 Hz) and the filtering done also removed the 50-Hz noise that comes from electronic/electric devices in the country where the data were collected.

Something that is very common in many EEG meditation data analysis is to observe each data visually and remove some sub parts manually to get rid of faults, based on the wave appearance. This is highly questionable because the way one would visually observe faulty parts may be different from another and this is not scientific. Therefore, in this study, when a raw EEG data is selected for analysis, the whole data was taken for the study without removing any sub-part merely by visual observation. This allows another person to repeat the same procedure in future experiments.

Each selected EEG dataset was broken down into epochs of 2 s size with 1 s overlap. With a sampling rate of 1000, an epoch of 2 s will contain sufficient information for the feature extraction and classification. When an EEG data is broken into epochs, some information near to the splitting location gets lost. To avoid this, an overlapping of 1 s was used and this would put the separation location of one epoch placed in the middle of the adjacent epoch.

Here, two studies were conducted on the four mind tasks Pre-Resting, Post-Resting, LKM Self and LKM Others using the relevant EEG epochs. A pairwise comparison was done among these four mind tasks creating six pairs. They are (Pre-Resting/LKM Self), (Pre-Resting/LKM Others), (Pre-Resting/Post-Resting), (LKM Self/LKM Others), (Post-Resting/LKM Self) and (Post-Resting/LKM Other). These six pairs were used in single session study and in multi-session study where the results can be used to understand some characteristics of meditation EEG data.

The first study was conducted on the EEG data of 32 participants for a single session. A session consists of four mind tasks Pre-Resting, Post-Resting, LKM Self and LKM Others. Thus, six pairwise analysis was conducted. As an example, one pair is used in the explanation, as elaborated below, since the same procedure is applicable for all the six pairs. Moreover, the same procedure was conducted for all the 32 participants.

For a single person, the pair “Pre-Resting/LKM Self” was selected. Here, EEG data were read, filtered and broken in to two groups of epochs. Then the dataset was divided into training and testing sets where 70% was used for the training and 30% for testing. For the training and testing, a machine learning pipeline was built using Python scikit-learn library which consists of three steps. As the first step in the pipeline, a covariance matrix was estimated for the input data using Oracle Approximation Shrinkage (OAS). Then as the second step, common spatial pattern (CSP) with (nfilter = 8) was used for the feature extraction. Here, eight spatial filters were used in the CSP filter bank, while reducing the dimensionality of covariance matrix to extract the most relevant features. As the last step of the pipeline, linear discriminant analysis (LDA) was used as the classification algorithm. Then, training dataset was used for fitting with the pipeline to calculate the LDA coefficients that can be used to calculate the LDA scores for the testing data which will allow the classification of the test data. Then, the prediction accuracy for the test data was calculated using balanced_accuracy_score in Python. This was conducted 25 times and the average accuracy for the 25 tests for a single pair for a single person was calculated. A similar procedure was conducted for the remaining 5 pairs for the given person to calculate the prediction accuracies. This calculation was conducted on all selected 32 participants and the results are shown in Table 2.

**Table 2:** Average prediction accuracy (%) after conducting 25 tests each for 32 participants with single session using CSP and LDA (train 70%, test 30%)

Count	Participant no.	Pre-resting/LKM self	Pre-resting/LKM Others	Pre-resting/post-resting	LKM self/LKM others	Post-resting/LKM self	Post-resting/LKM other
1	1	100 ± 0	100 ± 0	100 ± 0	100 ± 0	100 ± 0	94.6 ± 3.4
2	2	100 ± 0	100 ± 0	100 ± 0	100 ± 0	100 ± 0	98.8 ± 1.8
3	3	100 ± 0	100 ± 0	100 ± 0	100 ± 0	81.9 ± 6.5	80.3 ± 4.4
4	5	100 ± 0	100 ± 0	100 ± 0	99.8 ± 0.6	99.7 ± 0.8	98.2 ± 1.8
5	6	100 ± 0	99.9 ± 0.1	100 ± 0	98.6 ± 1.2	100 ± 0	99.9 ± 0.1
6	7	100 ± 0	100 ± 0	100 ± 0	100 ± 0	100 ± 0	95.1 ± 2.8
7	8	99.3 ± 0.8	100 ± 0	100 ± 0	99.9 ± 0.4	97.2 ± 1.1	99.5 ± 0.6
8	11	99.4 ± 2.0	100 ± 0	100 ± 0	98.1 ± 1.6	99.7 ± 0.9	100 ± 0
9	12	98.6 ± 3.1	99.4 ± 0.3	100 ± 0	98.7 ± 2.2	100 ± 0	90.8 ± 6.1
10	13	98.6 ± 4.1	99.9 ± 0.2	99.7 ± 0.6	88.5 ± 6.6	94.5 ± 5.1	98.1 ± 1.7
11	14	100 ± 0	99.9 ± 0.3	100 ± 0	100 ± 0	99.7 ± 0.5	100 ± 0
12	17	97.5 ± 2.5	99.5 ± 1.0	98.4 ± 2.7	91.1 ± 5.3	99.2 ± 0.8	90.5 ± 4.6
13	18	99.8 ± 0.5	100 ± 0	100 ± 0	65.7 ± 8.5	92.4 ± 3.8	98.7 ± 2.0
14	19	100 ± 0	100 ± 0	99.7 ± 1.6	95.3 ± 4.8	97.7 ± 2.6	95.5 ± 4.6
15	21	100 ± 0	99.5 ± 1.7	100 ± 0	79.6 ± 8.1	99.5 ± 0.6	99.3 ± 1.5
16	22	100 ± 0	100 ± 0	100 ± 0	99.0 ± 0.7	96.9 ± 3.9	94.1 ± 6.4
17	23	100 ± 0	100 ± 0	100 ± 0	100 ± 0	100 ± 0	99.3 ± 1.1
18	24	100 ± 0	100 ± 0	100 ± 0	97.8 ± 2.5	97.3 ± 2.1	93.1 ± 4.0
19	25	100 ± 0	99.1 ± 1.2	99.8 ± 0.5	82.2 ± 8.5	98.4 ± 1.3	80.4 ± 7.3
20	28	98.1 ± 2.2	99.3 ± 1.0	97.9 ± 1.7	99.9 ± 0.1	94.6 ± 2.4	99.4 ± 1.4
21	29	98.2 ± 2.7	100 ± 0	100 ± 0	99.1 ± 1.7	99.2 ± 0.5	84.8 ± 3.5
22	33	97.8 ± 1.6	100 ± 0	100 ± 0	87.9 ± 6.9	88.8 ± 7.4	96.0 ± 4.6
23	36	98.5 ± 0.7	100 ± 0	100 ± 0	98.7 ± 0.8	100 ± 0	100 ± 0
24	37	100 ± 0	100 ± 0	100 ± 0	100 ± 0	100 ± 0	74.4 ± 5.3
25	38	100 ± 0	100 ± 0	100 ± 0	99.4 ± 1.1	100 ± 0	100 ± 0
26	39	95.1 ± 2.1	100 ± 0	100 ± 0	87.3 ± 6.5	100 ± 0	100 ± 0
27	40	99.9 ± 0.2	100 ± 0	99.9 ± 0.3	93.7 ± 4.7	98.4 ± 2.4	93.1 ± 3.1
28	41	99.9 ± 0.6	99.9 ± 0.4	99.6 ± 1.2	99.8 ± 0.9	100 ± 0	99.9 ± 0.5
29	42	99.8 ± 0.4	99.9 ± 0.3	99.4 ± 1.3	99.6 ± 1.0	98.8 ± 0.5	85.7 ± 3.8
30	43	100 ± 0	100 ± 0	100 ± 0	99.4 ± 1.0	99.9 ± 0.3	99.2 ± 1.2
31	44	100 ± 0	92.3 ± 4.3	99.1 ± 1.2	98.5 ± 0.7	99.2 ± 0.5	99.6 ± 0.4
32	45	98.4 ± 2.4	100 ± 0	100 ± 0	100 ± 0	100 ± 0	99.1 ± 1.1
Mean accuracy		99.3	99.6	99.8	95.6	97.9	94.9

The second study was conducted on the EEG data collected from the 15 participants for multiple sessions where each session consists of four mind tasks Pre-Resting, Post-Resting, LKM Self and LKM Others. Here, per person five sessions were selected (Table 1) for the study of behavior of EEG meditation data for multiple sessions. In this test, prediction accuracy was calculated for each person for all six mind task pairs. They are (Pre-Resting/LKM Self), (Pre-Resting/LKM Others), (Pre-Resting/Post-Resting), (LKM Self/LKM Others), (Post-Resting/LKM Self) and (Post-Resting/LKM Other). When considering a single pair such as Pre-Resting/LKM Self for a single person, corresponding EEG data for the five sessions were used. These EEG data for the five sessions for a given pair, were read, filtered, broken down into epochs with the labels of the two mind tasks. In this case, one group will have epochs of Pre-Resting EEG data of five sessions and the other group will have epochs of LKM Self EEG data of five sessions. Then the dataset was divided into training and testing sets where 70% was used for the training and 30% for testing. Similar to the first study (above-mentioned), a machine learning pipeline with CSP and LDA was used for feature extraction and classification and the average prediction accuracy for 25 experiments was calculated for the test dataset. This was repeated for all six pairs for a single person and the calculation was conducted on all 15 participants and the results are shown in Table 3. Using 70% of the data for training is the standard procedure, but there are cases where an algorithm may go for a bias training and produce a good accuracy when testing for the remaining 30% data. This kind of faulty training can be traced by reducing the training dataset size and increasing the testing dataset size and then comparing the accuracies for different training data sizes. One such case is displayed here, where the whole second experiment was repeated with the change where 20% of the data were used for the training and the remaining 80% were used for the testing and corresponding prediction accuracies are shown in Table 4.

**Table 3:** Average prediction accuracy (%) after conducting 25 tests each for 15 participants with 5 sessions using CSP and LDA (train 70%, test 30%)

Count	Participant no.	Pre-resting/LKM self	Pre-resting/LKM others	Pre-resting/post-resting	LKM self/LKM others	Post-resting/LKM self	Post-resting/LKM other
1	1	83.2 ± 3.0	89.3 ± 2.5	93.8 ± 1.9	74.7 ± 4.2	82.2 ± 2.9	80.8 ± 2.8
2	3	90.9 ± 4.4	93.7 ± 2.6	94.9 ± 3.2	67.5 ± 2.6	78.6 ± 2.0	74.2 ± 2.9
3	7	82.8 ± 1.6	80.1 ± 2.2	67.0 ± 4.8	79.4 ± 1.6	67.2 ± 1.7	73.4 ± 2.4
4	8	85.8 ± 2.2	86.4 ± 2.1	91.0 ± 1.6	85.0 ± 2.7	86.6 ± 2.0	76.5 ± 2.1
5	11	85.9 ± 4.7	84.8 ± 5.2	85.6 ± 3.2	79.9 ± 1.9	75.8 ± 2.9	84.8 ± 2.5
6	12	80.5 ± 2.1	88.1 ± 2.0	81.6 ± 2.2	86.2 ± 2.3	79.4 ± 7.4	84.0 ± 2.6
7	14	80.4 ± 2.2	79.8 ± 2.7	90.2 ± 3.7	63.9 ± 4.6	71.6 ± 4.0	75.9 ± 4.4
8	37	70.6 ± 2.2	78.8 ± 1.6	75.6 ± 3.9	72.7 ± 2.7	77.8 ± 1.4	62.0 ± 4.6
9	38	74.7 ± 2.1	89.2 ± 3.6	94.0 ± 2.2	79.0 ± 2.4	76.9 ± 4.2	87.9 ± 1.8
10	39	91.7 ± 3.5	91.2 ± 3.2	83.6 ± 7.0	78.3 ± 4.4	75.2 ± 5.0	78.4 ± 4.8
11	41	89.4 ± 2.2	72.8 ± 5.4	89.8 ± 3.0	87.5 ± 1.2	82.0 ± 2.2	77.2 ± 3.1
12	42	82.0 ± 3.4	83.9 ± 2.9	85.1 ± 2.8	65.0 ± 2.9	80.6 ± 4.6	69.7 ± 1.6
13	43	83.4 ± 5.1	83.6 ± 5.2	82.1 ± 2.5	76.9 ± 3.7	70.3 ± 3.8	69.7 ± 3.9
14	44	81.3 ± 4.3	73.1 ± 3.3	85.6 ± 4.3	77.8 ± 3.1	83.6 ± 2.2	85.0 ± 2.1
15	45	78.0 ± 2.9	90.5 ± 2.4	89.4 ± 4.0	75.6 ± 2.9	79.1 ± 6.4	79.1 ± 2.7
Mean accuracy		82.7	84.4	85.9	76.6	77.8	77.2

**Table 4:** Average prediction accuracy (%) after conducting 25 tests each for 15 participants with 5 sessions using CSP and LDA (train 20%, test 80%)

Count	Participant no.	Pre-resting/LKM self	Pre-resting/LKM others	Pre-resting/post-resting	LKM self/LKM others	Post-resting/LKM self	Post-resting/LKM other
1	1	79.7 ± 3.8	86.6 ± 2.7	90.7 ± 4.8	68.9 ± 4.3	82.2 ± 5.8	82.4 ± 2.8
2	3	91.2 ± 4.5	90.0 ± 4.0	92.7 ± 4.3	66.9 ± 4.2	79.4 ± 5.7	72.7 ± 6.8
3	7	82.0 ± 2.4	78.9 ± 3.1	68.2 ± 4.2	78.8 ± 2.5	66.7 ± 1.1	72.7 ± 2.3
4	8	86.2 ± 1.2	86.5 ± 1.6	90.8 ± 1.3	79.6 ± 5.3	84.6 ± 3.0	75.8 ± 3.4
5	11	80.8 ± 5.8	78.9 ± 5.2	86.6 ± 3.3	78.9 ± 1.4	79.3 ± 3.5	83.9 ± 1.3
6	12	80.2 ± 1.3	85.4 ± 3.9	81.1 ± 2.6	86.0 ± 2.9	81.5 ± 6.2	84.2 ± 2.5
7	14	78.9 ± 3.4	80.0 ± 2.0	88.4 ± 6.1	64.8 ± 4.0	72.2 ± 4.2	73.9 ± 6.6
8	37	68.5 ± 4.4	79.7 ± 1.6	75.9 ± 3.2	67.8 ± 3.7	76.5 ± 2.1	69.6 ± 6.4
9	38	78.1 ± 4.8	90.3 ± 3.6	91.0 ± 3.5	77.5 ± 2.9	80.8 ± 4.7	84.2 ± 4.8
10	39	90.6 ± 3.0	91.0 ± 2.7	81.8 ± 6.5	84.5 ± 6.6	75.6 ± 3.1	75.7 ± 3.9
11	41	89.7 ± 3.1	82.4 ± 7.3	89.5 ± 3.4	84.8 ± 2.9	84.8 ± 5.5	80.7 ± 4.8
12	42	79.7 ± 3.3	80.7 ± 4.0	85.2 ± 3.1	65.3 ± 3.2	75.7 ± 5.1	69.5 ± 2.4
13	43	83.2 ± 6.0	85.3 ± 5.9	79.7 ± 3.5	76.9 ± 4.9	68.9 ± 5.2	71.0 ± 4.7
14	44	83.2 ± 5.1	75.4 ± 6.9	86.7 ± 4.2	76.3 ± 4.1	74.7 ± 5.7	83.2 ± 3.4
15	45	78.6 ± 3.2	89.3 ± 1.4	90.0 ± 2.9	71.0 ± 5.1	80.9 ± 5.0	72.8 ± 5.9
Mean accuracy		82.0	84.0	85.2	75.2	77.6	76.8

## Results

The first target of this study was to see how well a meditation EEG data can be classified from a non-meditation EEG data. For that, data of two meditation techniques were used, thereby attempting to see how these two types of meditation mind tasks classify with the two non-meditation mind tasks. The two non-meditation mind tasks were labeled as Pre-Resting and Post-Resting. Pre-Resting is the first mind task where the person stays in rest without doing any meditation. This is followed by the second and the third mind tasks which are meditation mind tasks (LKM-Self, LKM-Others). Lastly this is followed by the fourth mind task (Post-Resting) where the person stays in rest without doing any meditation after finishing the two meditation mind tasks. Our first task was to study how well each non-meditation instance classify with each meditation technique. Moreover, the next step was to check how well two meditation types can be classified. Finally, how well the two non-meditation sessions (Pre-Resting and Post-Resting) can be classified was checked.

Each individual result in Tables 2–4 shows the average prediction accuracy for each participant for a given pair of mind tasks. The six mind task pairs are (Pre-Resting/LKM Self), (Pre-Resting/LKM Others), (Pre-Resting/Post-Resting), (LKM Self/LKM Others), (Post-Resting/LKM Self) and (Post-Resting/LKM Other). The average value for each instance was calculated by conducting 25 experiments and averaging the obtained prediction accuracies.

Table 2 contains the average prediction accuracy for 32 participants after conducting 25 tests per participant. Original dataset had 48 participants, from which 32 participants had usable EEG data which were used for the analysis. A machine learning pipeline with CSP and LDA was used to get the results where 70% of the data were used for the training and 30% for the testing. The last row of Table 2 shows the average prediction accuracy calculated for all 32 participants. Here a single prediction accuracy per each pair of mind tasks indicates the performance of the algorithms used as well as some characteristics of the EEG meditation dataset that is being studied here.

The average classification accuracy obtained for Pre-Resting/LKM Self is 99.3% and for Pre-Resting/LKM Others is 99.6%. This gives an average of 99.5% for classifying meditation/Pre-Resting instances. At the same time, Post-Resting/LKM Self and Post-Resting/LKM other obtained 97.9% and 94.9%, respectively. Thus, giving an average of 96.4% for classifying meditation/Post-Resting instances. Pre-Resting/Post-Resting classification has given a high accuracy of 99.8%, but LKM Self/LKM Others has given a slightly lower accuracy of 95.6%.

After obtaining a high classification accuracy for the single session case, the conditions were evaluated for multiple sessions. The purpose is to test if some similar characteristics are shared among multiple sessions for a selected mind task for a given person. For checking this, 5 sessions were selected and a pool of epochs for each mind task was created. Using this, the classification accuracy for two mind tasks was checked where data from 5 sessions were used. The results are shown in Tables 3 and 4. Table 3 demonstrates the results of accuracies obtained where 70% of the data have been used for training and the rest for the testing, whereas in Table 4, 20% of the data were used for training and 80% of the data for testing. With such a huge reduction in the training data, less than 1% reduction in the accuracy for five out of the six tests was observed. The remaining one has less than 1.5% reduction in the accuracy. This is remarkable because 20% training data out of five sessions will on the average have data size of one session. This implies that only few data are enough to do the classification without a heavy loss in accuracy.

Table 3 contains the average prediction accuracy for 15 participants after conducting 25 tests. Original dataset had 48 participants, from which 15 participants were selected for collecting EEG data for multiple sessions containing 8 to 10 sessions. For all these 15 participants, 5 or more sessions were in good readable condition. Therefore, among these good-quality EEG data sessions, the largest 5 sessions were used in this analysis. CSP and LDA algorithms were used for feature extraction and classification. Here, 70% of the data were used for the training and 30% for the testing. The last row of Table 3 shows the average prediction accuracy calculated for all 15 participants which shows the prediction accuracy per each pair of mind tasks. Here a single prediction accuracy per each pair of mind tasks indicates the performance of the algorithms used as well as some characteristics of the EEG meditation dataset.

Table 4 contains the average prediction accuracy for 15 participants after conducting 25 tests and this study differs from the study where the results are shown in Table 3 by only one condition, which is the training and testing dataset sizes. A machine learning pipeline with CSP and LDA was used to get the results where 20% of the data were used for the training and 80% for the testing.

Since Tables 3 and 4 have almost similar results, only one of them is explained here (Table 3). The multiple session classification was done using the data of 15 participants for 5 sessions. The results show that the pairs (Pre-Resting/LKM Self), (Pre-Resting/LKM Others) and (Pre-Resting/Post-Resting) have relatively higher accuracies compared to the other pairs (LKM Self/LKM Others), (Post-Resting/LKM Self) and (Post-Resting/LKM Other). Here the average classification accuracy obtained for Pre-Resting/LKM Self is 82.7% and for Pre-Resting/LKM Others is 84.4%. This gives an average of 83.6% for classifying meditation/Pre-Resting instances. At the same time, Post-Resting/LKM Self and Post-Resting/LKM Others obtained 77.8% and 77.2%, respectively. Thus, giving an average of 77.5% for classifying meditation/Post-Resting instances. Pre-Resting/Post-Resting classification has given a relatively high accuracy of 85.9%, but LKM Self/LKM Others has given a slightly lower accuracy of 76.6%. When comparing the first three pairs and the other three pairs an accuracy reduction of 5% – 8% can be observed.

**Fig. 1:**
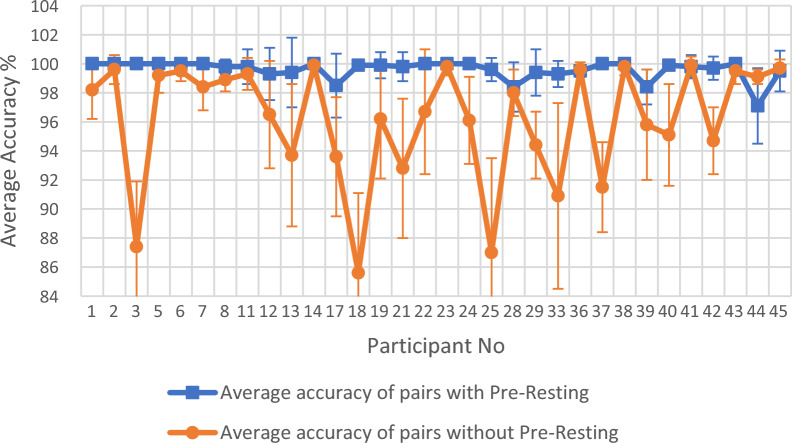
Comparison of average of first three accuracy columns (average accuracy of pairs with Pre-Resting) with average of last three accuracy columns (average accuracy of pairs without Pre-Resting) for the 32 participants in Table 2 for single session classification

**Fig. 2:**
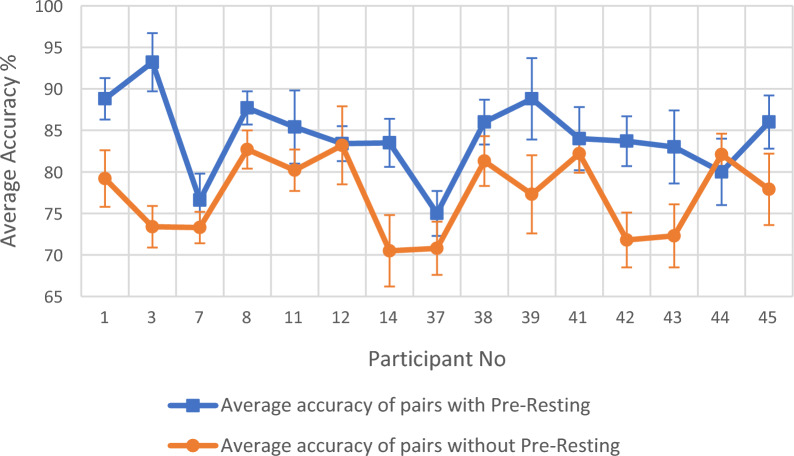
Comparison of average of first three accuracy columns (average accuracy of pairs with Pre-Resting) with average of last three accuracy columns (average accuracy of pairs without Pre-Resting) for the 15 participants in Table 3 for multiple session classification

Figure 1 gives the comparison plot of average accuracy difference between first three accuracy columns and last three accuracy columns for Table 2. This plot is for the single session classification using 32 participants. Figure 2 gives the comparison plot of average accuracy difference between first three accuracy columns and last three accuracy columns for Table 3. This plot is for the multiple session classification using 15 participants.

## Discussion

An EEG dataset collected from Loving Kindness Meditation for single and multiple sessions was used in this study and the dataset is currently available online. The most significant factor of the dataset is that it is a large dataset consisting of several meditation/non-meditation mind tasks collected for multiple sessions for multiple users. This gave the opportunity to study several patterns related to meditation EEG data for both single session and multiple sessions. The results we obtained in this study shows that good classification accuracy can be obtained for some instances, whereas for other instances this accuracy is slightly lower than the high accuracy ones, where all pairs were given similar analysis conditions. Based on these values we can understand some mind tasks have more similarities among them which make them hard to separate while the other mind tasks are easily separable owing to their differences. This is a valuable information that we can observe from the result set, which will be clearly explained below. At the same time, obtaining a very high accuracy for separating meditation and rest before meditation is also a high achievement in this study.

The ability to classify two pools of epochs from multiple sessions related to meditation mind tasks has a huge benefit, as explained in the introduction. With a 20% training data, a classification accuracy around 83% was obtained for the remaining 80% of the testing data (Table 4). This shows that some common characteristics are available among meditation multiple sessions. One such explanation we can give for this is we used 20% of the data taken from ten sessions where both groups contained five sessions each. Then, this 20% was used for the training and the remaining 80% from the ten groups were used for testing. Only 20% of training data and the large testing data (80%) produce an accuracy greater than 80% when doing the classification, thus giving the idea that some similarity is available among the five sessions of each group. Similarly, common characteristics are available among Pre-Resting multiple sessions. This indicates that if such similar characteristics could be identified for the current dataset, it is possible to use that knowledge to identify whether the new session is meditation or non-meditation. This paves the path for a future research opportunity where you use multiple sessions of meditation/non-meditation EEG data for training an algorithm and using that algorithm to determine into which category (meditation or non-meditation) a new mind task dataset will fall. At the moment, that is the biggest limitation in this study since you can only use data from the pool of epochs for testing and this does not currently support EEG data from a new session determining if this new data is a meditation or if it is a non-meditation session.

### Understanding the EEG meditation dataset based on the results obtained

When considering the 4 mind tasks, Pre-Resting is the starting session where EEG data are collected before starting any meditation practice. On the other hand, Post-Resting is a resting session where EEG data are collected after finishing a meditation session. Pre-Resting and Post-Resting are not totally equal because as an example if a person’s mind gets calm when meditating, in the Post-Resting instance, immediately after a meditation session, this calmness might be still slightly lingering. This can be supported if Post-Resting has more similarities with the meditation sessions and also if it has some differences with Pre-Resting instance. LKM Self, LKM Others are two meditation sessions done in the middle in between Pre-Resting and Post-Resting instances. Where meditation loving kindness is done to oneself or others and the corresponding data were collected.

When looking at Tables 2–4, a pattern similarity in all 3 of them can be observed. For all three tables, when considering the six columns used for classification accuracies for the 6 pairs, the first 3 columns give higher accuracies compared to the remaining 3 columns. This difference is 2–4% for Table 2. But in Tables 3, 4, the difference has increased up to 5–8%. Here, the prediction accuracy difference was obtained while using similar conditions for all instances including the feature extraction and classification algorithms. Although the method of analysis is similar for all the six pairs, they exhibit two levels of accuracies. This implies the following facts. Firstly, a higher prediction accuracy between a pair indicates there is a larger difference between the two and a smaller similarity between the two. On the other hand, for a pair, if the prediction accuracy has reduced it means that there is a smaller a difference and a larger similarity between the two in the pair, when comparing with a higher prediction accuracy.

The classification accuracy difference between first three accuracy columns and last three accuracy columns of Tables 2 and 3 are clearly demonstrated in Figs. 1 and 2. Figure 1 contains the summary of the single-session 32-participant accuracy data shown in Table 2. Here, for each participant ‘Average accuracy of pairs with Pre-Resting’ was calculated using the first three accuracy columns of Table 2 and ‘Average accuracy of pairs without Pre-Resting’ was calculated using the last three accuracy columns of Table 2. The results show that out of the 32 participants, 28 participants have a higher accuracy average for the first three accuracy columns compared with the last three accuracy columns in Table 2. Among these 4 remaining participants, 3 of them have a marginal difference and only the participant no. 44 falls out of this pattern with a big value. The 28 participants out of 32 in Fig. 1 clearly indicate that the first three column average accuracy is larger than the last three column average accuracy in Table 2. This observation is further proven by Fig. 2. Figure 2 contains the summary of the multiple session 15 participant accuracy data shown in Table 3. Here, for each participant ‘Average accuracy of pairs with Pre-Resting’ was calculated using the first three accuracy columns of Table 3 and ‘Average accuracy of pairs without Pre-Resting’ was calculated using the last three accuracy columns of Table 3. The results show that out of the 15 participants, 14 participants have a higher accuracy average for the first three accuracy columns compared with the last three accuracy columns in Table 3. The only participant that deviated from this pattern was participant no 44, which is the same participant that gave a significant difference if Fig. 1. The 14 participants out of 15 in Fig. 2 clearly indicate that first three column average accuracy is larger than the last three column average accuracy in Table 3.

The results suggest that the 3 pairs; (Pre-Resting/LKM Self), (Pre-Resting/LKM Others) and (Pre-Resting/Post-Resting) have produced higher classification accuracies. The first two pairs compare Pre-Resting instance with a two meditation instances. This proves that a higher classification accuracy can be achieved for a Pre-Resting instance and a meditation session. On the other hand, the higher classification accuracy between Pre-Resting/Post-Resting indicates that there is a higher difference between the two instances.

In terms of the last two columns in the three tables, we can see that in both instances it exhibits lower accuracies compared to first three accuracy columns. This indicates that when comparing EEG of each meditation and Post-Resting instance, they have more similarity than comparing with Pre-Resting and meditation instances. At the same time the pair LKM Self/LKM Others, having a low classification accuracy, indicates both these meditation techniques have some shared characteristics.

Concluding remarks for Tables 2–4, we can observe that the instance Pre-Resting, when comparing with LKM Self, LKM Others and Post-Resting, gives the highest classification accuracies. This shows that Pre-Resting has a big difference from the other three. In the pairwise comparison among LKM Self, LKM Others and Post-Resting only a lower classification accuracy is obtained. This indicates that the difference among these three is low and the similarity is high when compared with Pre-Resting; suggesting that the EEG collected for the two meditation sessions and the Post-Resting session are almost similar to each other.

Meditation may cause certain significant changes in a person including the EEG data patterns. This is divided into two types namely ‘states’ and ‘traits’ [20]. ‘States’ are temporary changes happening when meditating compared to the rest instance before beginning the meditation. ‘Traits’ are some permanent changes that happens to a person doing meditation for a long time. The high EEG difference mentioned above, between the Pre-Resting instance and a meditation session (LKM Self or LKM Others) equals to a ‘states’ change. The high EEG difference between Pre-Resting and Post-Resting indicates a significant difference between the two. Furthermore, the similarity (low difference) between Post-Resting and a meditation session (LKM Self or LKM Others) indicates that they have some common characteristics. But the difference between Pre-Resting and Post-Resting is not a ‘trait’ change and it is a ‘state’ change because it is more of a temporary relaxation happening in a person after a meditation session.

### Limitations of the study due to dataset characteristics

The dataset used in this study is of good-quality EEG data which we obtained after cleaning the original dataset and we selected four mind tasks for the analysis. The dataset was originally collected using 45 participants for one session and out of which 15 participants were used to collect multiple sessions. But one significant limitation of the collected dataset is, for the 45 participants the level of experience of meditation had not been collected as a measurement. The participants are said to be experienced meditators, but the level of experience of doing meditation is missing. Because of this lack of information, we can only study the state characteristics of the participants and we cannot study the trait characteristics of the participants related to the meditation tasks. Hence, one significant limitation of the dataset is, we can only study state characteristics and not the trait characteristics.

## Conclusion

This study was conducted to classify EEG data on four mind tasks, namely two types of meditation, pre-resting before meditation and post-resting after meditation using CSP and LDA as the classification algorithms. The results show that CSP and LDA combination was very successful in classifying a single session EEG data on meditation and Pre-Resting mind task (average accuracy = 99.5%). For the multiple session instance, although CSP and LDA does some decent classification even for a 20% training data, further research needs to be done to increase the classification accuracy.

Relatively higher classification accuracies were obtained for the pairs (Pre-Resting/LKM Self), (Pre-Resting/LKM Others) and (Pre-Resting/Post-Resting) when comparing with the pairs (LKM Self/LKM Others), (Post-Resting/LKM Self) and (Post-Resting/LKM Other). The results indicates that Pre-Resting has a larger difference compared with Post-Resting, LKM Self and LKM Others. On the other hand, among Post-Resting, LKM Self and LKM Others there seems to be a lesser difference.

The results show the hidden characters among EEG data of meditation-related mind tasks. These hidden characters are vital in research endeavor involving developing useful algorithms that can help to get a better picture of a new meditation mind task when compared with previous mind tasks.

## Data Availability

The dataset DOI is "https://doi.org/10.18112/openneuro.ds003816.v1.0.1" and is available at “https://openneuro.org/datasets/ds003816/versions/1.0.1”.
